# Retinal Degeneration Protein 3 (RD3) in normal human tissues: Novel insights

**DOI:** 10.1038/s41598-017-13337-9

**Published:** 2017-10-13

**Authors:** Sheeja Aravindan, Dinesh Babu Somasundaram, Kwok Ling Kam, Karthikeyan Subramanian, Zhongxin Yu, Terence S. Herman, Kar-Ming Fung, Natarajan Aravindan

**Affiliations:** 1 Stephenson Cancer Center, Oklahoma City, OK USA; 2 Departments of Radiation Oncology, University of Oklahoma Health Sciences Center, Oklahoma City, OK USA; 3 Pathology, University of Oklahoma Health Sciences Center, Oklahoma City, OK USA

## Abstract

The 195-amino-acid-long human Retinal Degeneration Protein 3 (RD3) is critical in the regulation of guanylate cyclase (GC) signaling and photoreceptor cell survival. Recently, we identified significant loss of RD3 in high-risk neuroblastoma and the influential role of RD3 in tumor progression. However, the functional characterization of RD3 in tumor systems has been hampered by the dearth of information on its localization in normal tissue and by the lack of antibodies suitable for staining FFPE tissue, primarily due to the inaccessibility of the epitopes. In this study, we validated a custom-synthesized RD3 antibody and investigated the expression/localization of RD3 in assorted human tissues. We observed stratified expression of RD3 in different cell types and subcellular location of retina. We demonstrated extensive positive RD3 immunoreactivity in various normal tissues and particularly strong dot-like perinuclear staining in the lining epithelial cells, suggesting that RD3 may play an important role in the normal functioning of epithelial cells. RD3 expression is limited in the CNS. While neuroblastoma is often RD3-positive, the adrenal medulla, where many neuroblastomas originate, is RD3-negative. Meta-analysis of RD3 transcriptional expression across normal tissues confirmed tissue-specific RD3 mRNA levels. Our results revealed the tissue-specific expression/localization profile of RD3 for the first time.

## Introduction

Retinal degeneration protein 3 (RD3/LCA12/C1orf36) is a gene that encodes a 195-amino-acid-long protein with relatively low molecular mass (22 kDa) and includes putative coil-coil domains at amino acids 22–54 and 115–141 and several conserved sites for protein modification, and is expressed in rod and cone photoreceptor cells^1^. RD3 protein is highly conserved across vertebrates with the human protein, sharing 95% sequence identity with other primates, 86% with mice and rats, 83% with bovine animals, 67% with chickens, and 50–60% with lower vertebrates (zebrafish, Western clawed frog)^2^. Retinal degeneration studies showed that genetic defects or mutations in RD3 (e.g., homozygous c.319C → T in exon 3) produce a less stable non-functional C-terminal truncated protein that drives early-onset photoreceptor degeneration in patients with Leber Congenital Amaurosis 12^3^. Recent studies underscored the importance of RD3 in photoreceptor cell survival, and provided insight into the function of RD3 in photoreceptor cells, as well as the mechanism by which mutations in RD3 cause photoreceptor degeneration^1,2,4–6^. RD3 binds to guanylate cyclases GC1 and GC2, translocate GCs from the ER to the photoreceptor outer segments, and suppresses the basal enzymatic activity of GCs^1,2,4^. In addition, RD3 mice lack GC expression in the retina; this finding highlights the importance of RD3 in maintaining GC expression and stability^1^. Forced delivery of the normal *RD3* gene restores GCs expression and outer segment localization, and leads to the long-term recovery of visual function and photoreceptor cell survival^4^. Although high levels of RD3 expression in rod and cone photoreceptor cells and RD3’s association with photoreceptor cell survival have been extensively recognized, information on RD3 constitutive expression and/or localization in other tissue/cell types is limited. In the present study, we investigated the transcription and tissue-specific expression/localization of RD3 protein in various human tissues.

RD3 was primarily detected using mass-spectrometry-based proteomic analysis^7^. We and others have used immunoblotting to validate the presence of low molecular mass RD3 protein in tissue extracts with mono/polyclonal RD3 antibodies^1,8^. However, successful immuno-localization of RD3 in tissues is challenging and yields equivocal outcomes, with inconsistent labeling above background levels^1^. This is mainly due to the inaccessibility of the epitopes and/or the low level of constitutive or facultative RD3 expression in certain tissues. Establishing and characterizing an RD3-specific antibody that can access epitopes is needed. Thus, we custom-synthesized an anti-human RD3 antibody, characterized its specificity, and investigated the expression and localization of RD3 in several human tissues. Our immunohistochemical approach used an automated staining process in order to maintain the quality and minimize variation of staining results, in contrast to manual staining methods.

We demonstrated significant loss of RD3 (transcriptional/translational) in *in vivo* mouse models and in clinical samples of high-risk neuroblastoma^8^, the most common extracranial malignant solid tumor in infants and children. RD3 loss is strongly correlated with advanced stages of neuroblastoma and with poor patient survival in multiple cohorts. More importantly, RD3 loss is correlated with increased metastasis, and we demonstrated its novel ability to stabilize tumor evolution, underscoring RD3’s possible role in the switch from neuroblastoma with favorable prognosis to high-risk aggressive disease^8^. The biological significance of this highly conserved protein in normal human tissue and tumors other than neuroblastoma is largely unexplored. To that end, we investigated the localization and constitutive expression of RD3 protein in a collection of normal human tissues, and further compiled the *RD3* transcriptional profile in normal tissues to enhance our understanding of RD3 in human tumors other than neuroblastoma.

## Results

### Antibody validation

**Figure 1:**
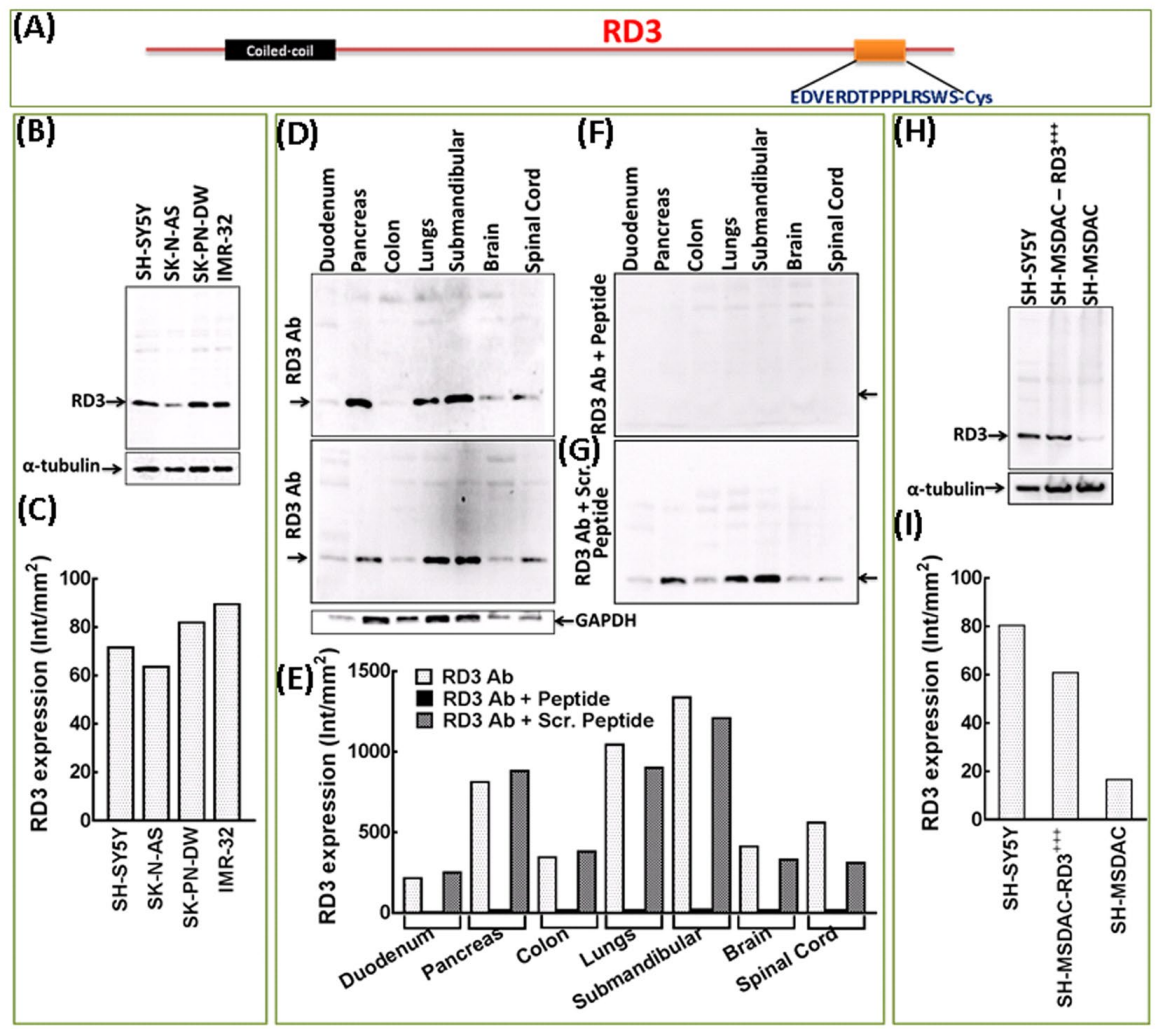
RD3 antibody validation. (**A**) Schematic representation of RD3 domain structure and the regions (amino acids 171-183) used for antibody production. Antibody was produced in collaboration with NeoBioLab (Cambridge, MA) on our initiative, affinity purified, and quality tested. (**B**) Immunoblot showing RD3 specific labeling by the custom synthesized antibody without any non-specific events. Cell lysates from human neuroblastoma (SH-SY5Y, SK-N-AS, SK-PNDW, IMR-32) cells blotted with synthesized RD3 antibody resulted in the labeling of single strong band at 23 kDa. (**C**) Histograms of Quantity One band ID gel analysis showing relative expression levels of RD3 in human neuroblastoma cell lines. Immunoblots showing RD3 levels in tissue lysates from human duodenum, pancreas, colon, lungs, submandibular gland, brain and spinal cord labelled with (**D**) custom synthesized RD3 Ab; (**F**) synthesized RD3 Ab neutralized with peptide (antigen) and; (**G**) synthesized RD3 Ab neutralized with scrambled peptide (SPDLRRESWDPVETP) containing same amino acid content. (**E**) Histograms from Quantity One gel densitometry analysis showing strong, tissue specific expression profile of RD3 with custom synthesized Ab; complete lack of labeling when the Ab is neutralized with peptide and; no non-specific blocking of custom Ab associated RD3 immunostaining when neutralized with scrambled peptide. (**H**) *RD3 specificity labeling defined with RD3 gene manipulation experiments*. Immunoblots showing corresponding, characteristic, RD3 labeling variations in SH-SY5Y (RD3 positive control), SH-MSDAC (RD3 negative control) and in SH-MSDAC-RD3^+++^ (ectopically re-expressed) cells lysates. (**I**) Histograms from Quantity One band intensity analysis reiterating high basal levels of RD3 in SH-SY5Y cells, loss of RD3 in SH-MSDAC and re-expression of RD3 in SH-MSDAC after forced ectopic expression. Full-length blots are presented in Supplementary Figure S6.

The RD3 anti-human antibody was designed and produced by the NeoBioLab (Cambridge, MA) on our initiative, in a project that emphasized generating RD3 antibodies that would be appropriate for IHC and devoid of any epitope inaccessibility issues. A sequence specific for RD3 (amino acids 171-183) was chosen for recombinant protein production and for immunization (Fig. 1A). Initial quality control studies with analytical HPLC and MS ensured the delivery of high-quality peptides for antibody production (Figure S1). The resultant rabbit polyclonal antibody was affinity purified and validated by ELISA analysis. Compared with blank and negative controls, ELISA revealed the RD3 antibody specificity in a concentration-dependent manner (1:1000, 4000, 16000, 64000), with definite sensitivity even with maximal (1:64000) dilution (Figure S1).

Immunoblot analysis was performed in several human neuroblastoma SH-SY5Y, SK-N-AS, SK-PNDW, and IMR-32 cells, as we have previously reported basal levels of RD3 expression in these cells^8^. Cell lysates blotted with the synthesized RD3 antibody contained a single 23-kDa band, indicating that the antibody specifically detected RD3 (Fig. 1B,C), and served as the positive control. More importantly, immunoblot analysis performed utilizing the lysates of normal human duodenum, pancreas, colon, lungs, submandibular gland, brain and spinal cord tissues with the custom synthesized RD3 antibody produced a single solid 23-kDa band, demonstrating the Ab specificity (Fig. 1D). Evidently, band intensity analysis demonstrated tissue specific RD3 expression profile that corroborates with the IHC data (Fig. 1E). Further antibody neutralizing experiments with pre-mixing antibody with antigen (peptide) showed complete loss of RD3 labeling, defining the antibody-specific labeling in the immunoblots of lysates from normal human duodenum, pancreas, colon, lungs, submandibular gland, brain and spinal cord tissues (Fig. 1F). The blots were over exposed to define the complete lack of RD3 labelling with the neutralized Ab (Fig. 1E,F). The appearance of the faint bands on the top of the blot (despite perfect absence of RD3 labeling) in this setting is consistent with the other full length blots (*in vitro* and in human tissues) and serves as the negative control. In addition, to ensure the specificity of the peptide antigen competition, the alterations in RD3 labeling (and levels) were investigated with the blots of lysates from identical set of human tissues were immunostained with neutralized Ab with the scrambled sequence peptide containing same amino acid content (Fig. 1G). Immunoblotting revealed no non-specific neutralization with scrambled peptide (Fig. 1G) and, the band intensity quantification revealed a near identical expression profile of RD3 (*vs*. non-neutralized Ab) in these tissues (Fig. 1E). Furthermore, we investigated the RD3 antibody specificity with RD3 gene manipulation experiments utilizing SH-SY5Y (with high basal level of RD3) and SH-MSDACs (RD3 null/lost) cells^8,9^. Immunoblotting with custom synthesized RD3 Ab reiterates RD3 expression levels of SH-SY5Y and SH-MSDAC cells (Fig. 1H,I). More importantly, immunoblotting revealed strong RD3-specific labeling in ectopically RD3 re-expressed MSDACs (Fig. 1H,I). The results from the RD3 gene silencing and re-expression studies coupled with immunoblotting analysis demonstrate the RD3 specificity of the antibody produced in the present work.

To further validate the antibody’s ability and specificity of RD3 labeling in FFPE tissues, we investigated RD3 labeling in human colon tissues, with and without neutralization, using various concentrations of premixed antibody + peptide. Automated IHC with no primary antibody controls in colon tissues produced no labeling, no background, and served as the negative control (Figure S2). However, IHC performed with RD3 antibody produced selective and specific RD3 labeling in human colon tissues (Figure S2). RD3 positive staining appeared in brown, and was predominantly localized to the nucleus and perinuclear area, with weak-to-moderate cytoplasmic positivity (Figure S2, 20X). Moreover, we observed that the antibody could differentiate sub-cellular selectivity (positive, negative), intensity (strong, moderate and weak positivity), and specific RD3 localization in an FFPE setting. RD3 antibody neutralizing protocols (i.e., pre-mixing with 1, 2, or 4 μg of antigen) completely blocked RD3 staining with as little as 1 μg antigen (Figure S2), demonstrating that the IHC staining was antibody-specific. Though, the use of RD3 mice with natural knockout would further be an ideal platform to evaluate the Ab specificity, since the synthesized RD3 Ab is anti-human, such an approach is not attempted in this study.

Finally, to irrefutably demonstrate the specificity of synthesized RD3 antibody and to portray the relative abundances of RD3 expression in human normal tissues, we investigated the RD3 labeling efficiency and expression levels in colon, pancreas, submandibular and duodenum and, compared with human retina, the only tissue for which the RD3 presence and its biological function has been documented thus far (Figure S3A,B). Retinal tissue lysates blotted with the synthesized RD3 antibody contained a single 23-kDa band without any cross reactivity, indicating that the antibody specifically detected RD3 (Figure S3A), and served as the positive control. More importantly, we observed a consistent single solid 23-kDa band with the lysates of duodenum, pancreas, colon and submandibular gland demonstrating the Ab specificity (Figure S3A). Evidently, band intensity analysis demonstrated the abundance of RD3 expression in retinal tissues (Figure S3B). Further RD3 expression was relatively high (near comparable to retinal expression) in submandibular tissue, moderate in pancreas, colon and duodenum (Figure S3B). These results clearly portray the RD3 specific labeling of the synthesized antibody and the relative abundance of RD3 expression in normal human tissues compared to its expression in RD3.

### RD3 transcription in human tissues

**Figure 2:**
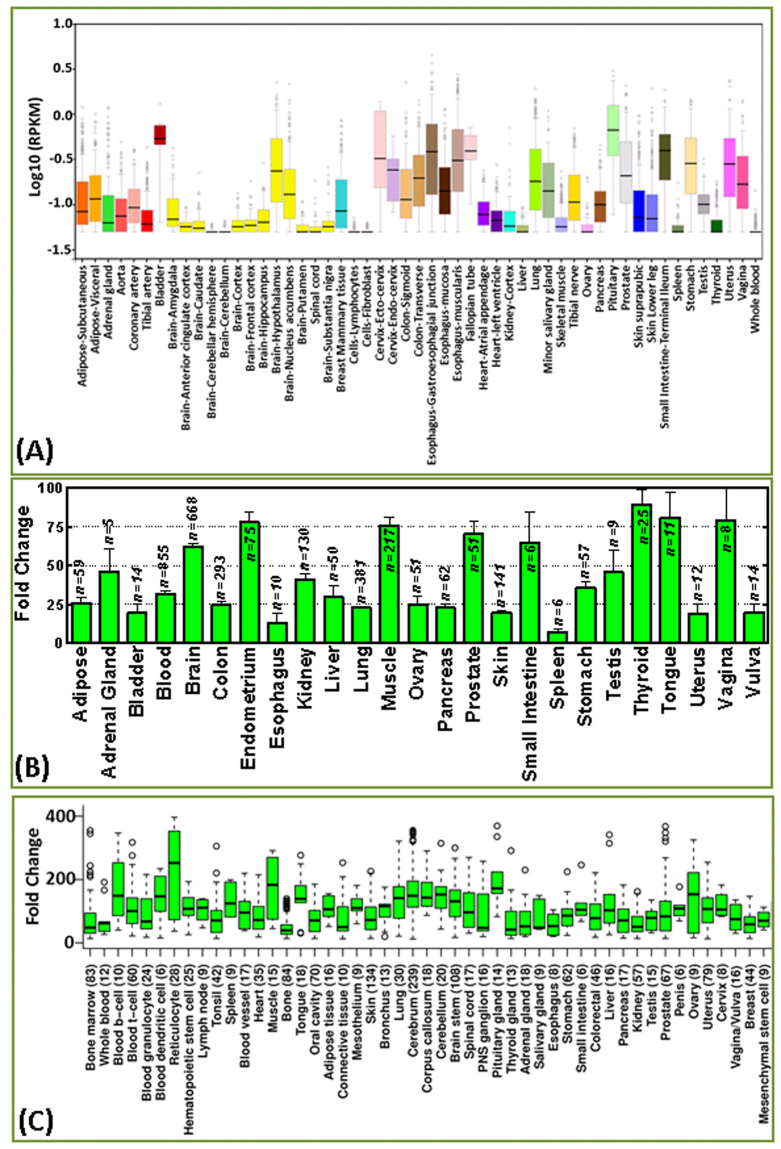
RD3 mRNA expression profile in normal human tissues. Histograms from *in silico* data analysis of RD3 mRNA expression from the (**A**) Genotype-Tissue Expression (GTEx), (**B**) Gene Expression across Normal and Tumor tissue, (GENT), and (**C**) Medisapiens *in silico* transcriptomics (IST) online public databases. The GTEx database included the analysis of 53 healthy tissues (total *n* = *8232*) on the Affymetrix and Illumina platforms, expressed in calculated RPKM with isoforms collapsed to single gene with no other normalization steps. The GENT database contained expression analysis in 25 healthy human tissues (total *n* = 3210) from the Affymetrix platform. The IST database included data analysis of gene expression from the Affymetrix platform across 49 healthy tissues (total *n* = 1706) with unique normalization and data quality verifications, allowing the gene expression profiles collected from different studies to be combined to generate an overview of the expression profile in human tissues. The expression profiles of RD3 mRNA levels in over 80 tissue types across three data portals corroborated well with each other, despite some intensity variations, and clearly identified the tissue-specific RD3 mRNA levels in normal human tissues.

*In silico* analysis revealed tissue-type-specific RD3 transcription in human tissues. Using data from three individual databases (GTEx, GENT, and IST), we compiled the RD3 mRNA levels in more than 80 tissue types, including adipose (adipose, subcutaneous, visceral), adrenal gland, bladder, blood vessel (blood vessel, aorta, coronary artery, tibial artery), bone, bone marrow, brain (brain, cerebrum, cerebellum, corpus callosum, brain stem, amygdala, anterior cingulate cortex, caudate, cerebella hemisphere, cortex, frontal cortex, hippocampus, hypothalamus, nucleus accumbens, putamen, substantia nigra), breast, bronchus, cells (lymphocytes, fibroblasts, blood B-cells, blood T-cells, blood-granulocytes, blood-dendritic cells, blood-reticulocytes, hematopoietic stem cells), cervix (cervix, ecto-cervix, endo-cervix), colon (colon, sigmoid, transverse), colorectal, connective tissue, endometrium, esophagus (esophagus, gastro-esophageal junction, esophagus mucosa, esophagus muscularis), heart (heart, atrial appendage, left ventricle), fallopian tube, kidney (kidney, kidney cortex), liver, lung, lymph node, mesenchymal stem cell, mesothelium, muscle (skeletal muscle), oral cavity, ovary, pancreas, penis, pituitary gland, prostate, salivary gland, skin (skin, suprapubic, lower leg), small intestine (SI, SI-terminal ileum), spinal cord (spinal cord, PNS ganglion), spleen, stomach, testis, thyroid, tibial nerve, tongue, tonsil, uterus, vagina and vulva (Fig. 2A–C). Considering the differences in the analyzed tissue types and analyzing platform/method across the databases, a thorough, careful comparison of RD3 mRNA expression across healthy tissues is warranted, and is presented below.

*In silico* transcriptomic data analysis indicated near-zero RD3 mRNA levels in lymphocytes and fibroblasts (Fig. 2A). Moderate RD3 transcription was observed in human brain tissue (Fig. 2B). Further, hypothalamus, nucleus accumbens, amygdala (Fig. 2A), cerebrum, corpus callosum, cerebellum, brain stem and tibial nerve (Fig. 2C) tissues showed strong RD3 transcription levels. However, anterior cingulate cortex, caudate, cortex, frontal cortex, hippocampus (Fig. 2A), spinal cord, and PNS ganglion tissues showed meagre RD3 transcription. Similarly, adipose tissue showed moderate (Figure A-C) RD3 levels, clearly indicating inter-adipose tissue variations with relatively high levels in visceral adipose tissue compared with low levels in subcutaneous adipose tissues. There was extensive variation between data sources regarding RD3 transcription levels in the adrenal gland, ranging from marginal to high levels. Bladder tissues showed fluctuating moderate to high levels of RD3 transcripts (Fig. 2A,B). Although RD3 analysis in whole blood showed near-zero levels in two databases (Fig. 2A,C), analysis of the outsized collection (*n* = 855) of samples revealed fairly high RD3 transcription (Fig. 2B) that was consistent with the high RD3 levels analyzed individually in B-cells, T-cells, granulocytes, dendritic cells, reticulocytes, and hematopoietic stem cells (Fig. 2C).

RD3 transcription in blood vessels varied with the type of vessel, including low transcription in tibial artery tissue, moderate transcription in the aorta, and high transcription in coronary artery tissue. Although basal levels of RD3 transcription were observed in heart tissues, the data analysis revealed markedly high RD3 transcription in the atrial appendage compared with the left ventricle (Fig. 2A,C). Moderate RD3 transcription levels were observed in the esophagus; however, transcription increased in the gastro-esophageal junction, mucosa, and muscularis. Despite minimal RD3 transcripts reported in muscle tissues in one database, the other databases consistently indicated high levels of RD3 in muscle tissues. Bone possessed insignificant levels of RD3. In contrast, tissues along the gastro-intestinal tract showed maximal levels of RD3 transcription. For example, small intestine, stomach, and colon (sigmoid, transverse) tissues showed overall high levels of RD3. RD3 was moderately expressed in pancreas tissues. Furthermore, levels of RD3 transcription fluctuated from moderate to high among tissues along the reproductive system, including the fallopian tube, ovary, endometrium, uterus, cervix (ecto-cervix and endo-cervix), vagina, and vulva (Fig. 2A–C). This moderate-to-high fluctuation in RD3 transcription was also apparent in male reproductive tissues (testis, penis, prostate; Fig. 2A–C). Analysis of skin tissues revealed trivial, yet measurable, RD3 transcription across databases without any tissue-origin- (supra-pubic, lower leg or others) specific fluctuations (Fig. 2A–C). Spleen, kidney, and liver tissues showed nominal to moderate RD3 transcription. However, RD3 level was relatively high in lung tissues. Similarly, a remarkably high level of RD3 was observed in tongue tissues, although tonsil and oral cavity tissues exhibited nominal RD3 transcription. Interestingly, data analysis of thyroid tissues unveiled equivocal outcomes (fluctuations from minimal to maximal RD3) with data sources. Tissues like lymph nodes, bone marrow, mesothelium, connective tissue, breast, and mesenchymal stem cells showed nominal expression (Fig. 2C). Salivary gland tissues showed moderate to high RD3 levels. Taken together, our analysis of RD3 transcription levels utilizing three high throughput data sources of numerous clinical tissues: (i) identified the basal levels of RD3 transcription in normal human tissues, (ii) recognized the inter- and intra-tissue-specific fluctuations in RD3 transcription, and (iii) defined the system-specific (e.g., GI, female reproductive system, CNS) association of RD3 transcription intensity. Although intensity variations were observed between the databases, the expression profiles generally complemented each other and hence allowed us to clearly define the RD3 transcription in normal human tissues. This *in silico* data analysis demonstrated the general pattern of RD3 transcription in normal human tissues. It should be noted that these data were generated from homogenized tissue without specific reference to a certain cell type. The expression levels of RD3 in different cell types could not be resolved using this technique.

Further to define the transcriptional levels of RD3 in normal tissues in comparison with human retina (that is not included in the data bases utilized in this study), the only tissue with documented abundance of RD3, we investigated the relative abundance in the transcript levels of RD3 in human retina, colon, pancreas, submandibular, lungs and duodenum (Figure S3C). QPCR analysis revealed relatively high levels of RD3 mRNA in human retina, submandibular and pancreas while we observed moderate RD3 levels in duodenum, lungs and colon. Together these results in general portray the abundance of RD3 transcripts in human normal tissues and further indicate the relative expression patterns of RD3 in colon, pancreas, submandibular, lungs and duodenum compared to the retinal expression (Figure S3C).

### RD3 protein expression and cellular localization in normal human tissues

**Figure 3:**
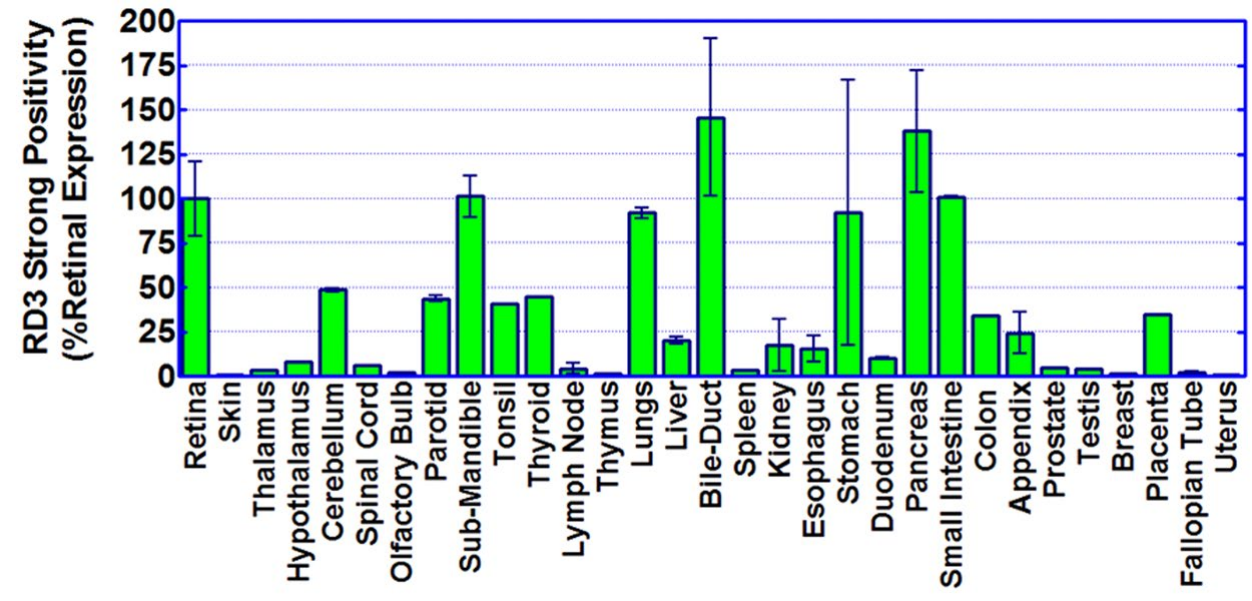
RD3 protein levels in normal human tissues. Histograms obtained from Aperio image analysis and quantification showing levels of RD3 strong positivity in human retina, skin, thalamus, hypothalamus, cerebellum, spinal cord, olfactory bulb, parotid, sub-mandible, tonsil, thyroid, lymph node, thymus, lungs, liver, bile duct, spleen, kidney, esophagus, stomach, duodenum, pancreas, small intestine, appendix, colon, prostate, testes, breast, placenta, fallopian tube, and uterus tissues. Automated RD3 IHC-stained sections of FFPE specimens were micro-digitally scanned using Aperio ScanScope, and RD3 strong positivity was group-analyzed using Aperio image analysis/quantification software. Tissue-specific expression was profiled and the retinal expression normalized (% retinal strong positivity). Mean and *SD* are plotted with GraphPad Prism.

Since RD3 expression is thus far unknown in normal human tissues other than the retina and is critical to defining its role in cancer biology, we examined the expression and localization of RD3 protein in the retina, central nervous system (brain, spinal cord, and olfactory bulb), gastrointestinal tract (esophagus, stomach, duodenum, appendix, colon), pancreatic hepatobiliary tract (parotid and submandibular gland, liver, bile ducts, pancreas), and other organs (lung, kidney, placenta, uterus, thymus, prostate skin, fallopian tube, thyroid, tonsil, breast). Our automated IHC coupled with Aperio image analysis and RD3 positivity scoring revealed strong positive RD3 staining in human retina that served as the positive control. Isotype matched controls in normal human tissues including retina, did not reveal any specific staining and hence served as the negative controls (Figure S4). Since RD3 is localized in both the nucleus and cytoplasm, immunoreactivity in both subcellular locations was counted together. Further, to avoid any false positive staining, only strong positivity signal quantification was included in this analysis. All calculated intensities were normalized to percent baseline (retinal expression) and expressed as means with standard deviations (Fig. 3). Overall, we observed nominal RD3 protein expression in human skin, thalamus, olfactory bulb, thymus, spleen, breast, fallopian tube, and uterus tissues (Fig. 3). Hypothalamus, spinal cord, lymph node, duodenum, prostate, and testis tissues exhibited low, yet measurable, RD3 expression. Human liver, kidney, esophagus, colon, appendix, and placenta tissues showed moderate (near 25% of retinal positivity) RD3 expression (Fig. 3). We observed strong (about 50% of retinal positivity) RD3 expression in cerebellum, parotid, tonsil, and thyroid tissues. Interestingly, we noted prominent RD3 protein expression (comparable to and beyond that of retinal positivity) in human submandible, lungs, bile-duct, stomach, pancreas, and small intestine tissues (Fig. 3). RD3 protein expression data for skin, cerebellum, spinal cord, thyroid, lungs, liver, spleen kidney, esophagus, stomach, and small intestine tissues corroborated well with the *in silico* transcription data (Fig. 2).

#### Retina, central nervous system, and adrenal gland

**Figure 4:**
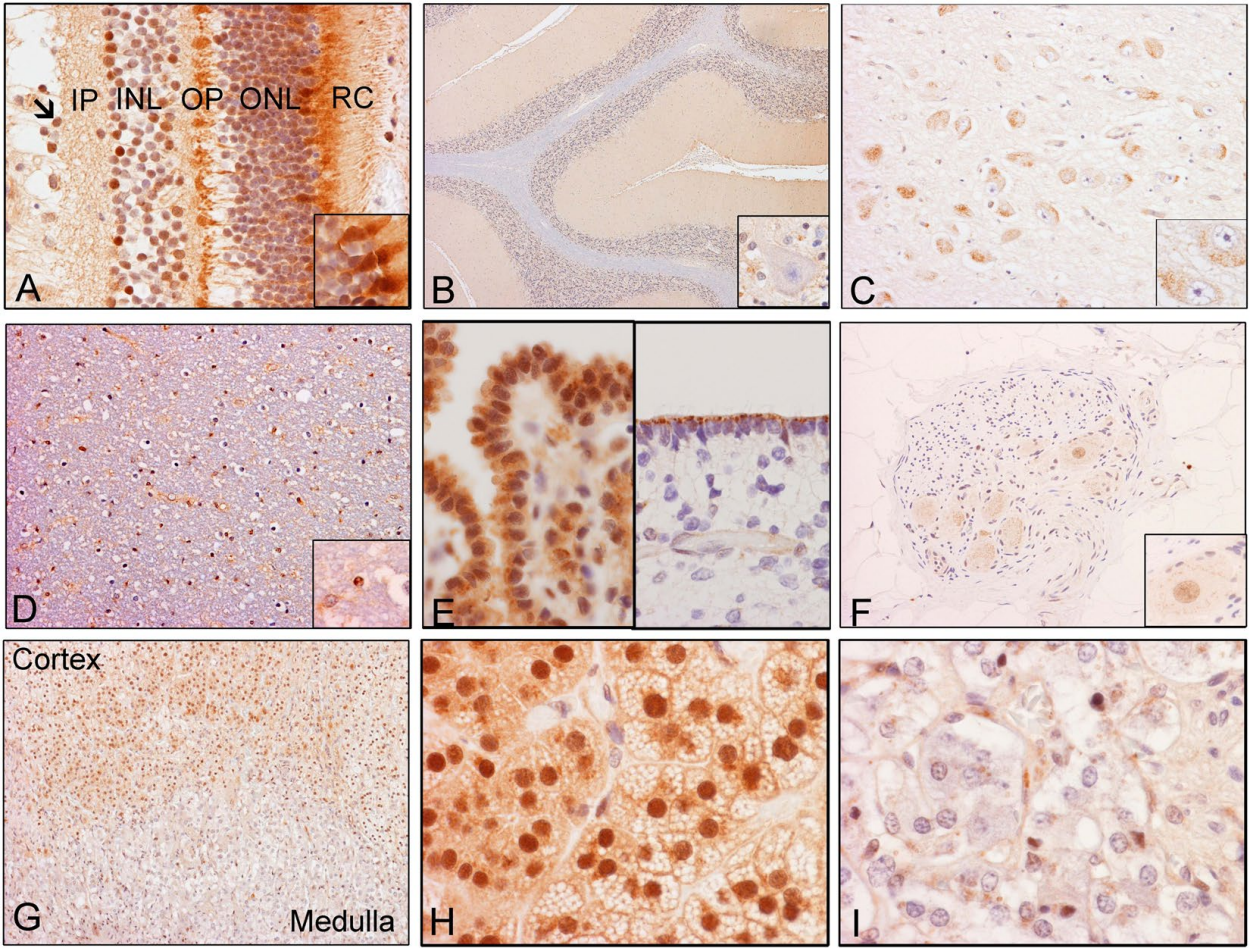
RD3 cellular localization in human tissues. Representative microphotographs showing RD3 expression and cellular localization in human (**A**) retina, (**B**) cerebellum, (**C**) basal ganglia, (**D**) spinal cord, (**E**) thalamus, (**F**) peripheral nerve ganglion, and (**G**–**I**) adrenal gland (***H***, *adrenal cortex*; ***I***, *adrenal medulla*) tissues. [Magnification 10x; Insert, 60x] ***IP*** = *inner plexiform layer*; ***INL*** = *inner nuclear layer*; ***OP*** = *Outer plexiform layer*; ***ONL*** = *outer nuclear layer*; ***RC*** = *Rods and Cones* (*cytoplasmic portion*).

Results are summarized in Table 1 and illustrated in Fig. 4. In the retina, the strongest immunoreactivities were located in the internal half of the photoreceptor layer and the external half of the outer plexiform layer. Both nuclear (arrow in Fig. 4A) and cytoplasmic immunoreactivities were observed. In the photoreceptor layer, some cells were more strongly reactive than others (insert in Fig. 4A). The distribution of immunoreactivities was not homogeneous, with the strongest immunoreactivity seen in the inner half of the cytoplasmic portion of the photoreceptor layer, where rods and cones are found, and the external half of the outer plexiform layer (Fig. 4A). However, to validate the specificity of custom synthesized Ab in detecting the localization of RD3 and, to demonstrate its heightened efficiency, human retinal sections were immunostained in parallel with commercially available RD3 Abs. For this we used a panel of 6 different Abs raised against different regions (AA 7-67; AA 36-85, AA 52-112, AA 62-87, AA 145-175, AA 135-194) of human RD3 protein. IHC analysis revealed RD3 staining pattern with varying degrees of magnitude in labeling efficiency, portraying the immaculate labeling efficiency of the custom synthesized Ab used in this study (Figs 4A and S4). Despite the differences in efficiency magnitude, the results clearly demonstrated the consistent distribution patterns of the RD3 localization as discussed above (Figure S5). In adult cerebellum (Fig. 4B), there was weak expression in the molecular layer of cerebellar folia, but the internal granular layer, Purkinje cells, and white matter were largely negative for RD3 (Fig. 4B). In adult cerebrum, there was moderate expression in the cytoplasm of large neurons, such as those in the thalamus, basal ganglia, and spinal cord (Fig. 4C). Neuropils in general were weakly negative; neuropils in the molecular layer of the cerebellum had the strongest expression. In the white matter, there were only scant positive nuclei, and no cytoplasmic positive immunoreactivity was noted (4D). In contrast, the cells lining the central nervous system, namely, the ependymal cells and choroid plexus epithelium (Fig. 4E), showed strong RD3 immunoreactivity. In the peripheral nerve ganglions, weak cytoplasmic reactivity was noted in the ganglionic neurons, but not in the sustentacular cells or Schwann cells (Fig. 4F). Some, but not all, of the nuclei of ganglionic cells were RD3-positive. In the adrenal gland (Fig. 4G to I), there were strong nuclear immunoreactivities and weak cytoplasmic immunoreactivities in the adrenal cortex (Fig. 4H). In contrast, adrenal medullar cells were largely negative for RD3 (Fig. 4I). Some positive cells were present in the adrenal medulla; these cells were more consistent with endothelial cells, but not adrenal medullar cells.

**Table 1:** RD3 localization in human tissues.

Nervous System and Adrenal Gland				
Tissue	Cell	Nuclear	Cytoplasm	Comment
Retina	Photoreceptor layer outer segment	N/A	●●●●●	NSP
	Photoreceptor layer inner segment	N/A	●●●●●	NSP
	Outer nuclear layer	●●●●		NSP
	Outer plexiform layer, outer half	N/A	●●●●	NSP
	Outer plexiform layer, inner half	N/A	●●●●●	NSP
	Inner nuclear layer	●●●●		NSP
	Inner plexiform layer	N/A	●●●●●	NSP
	Ganglion cell nuclei	●●●●	N/A	NSP
	Ganglion cell neuropil	N/A	●●●●●	NSP
Cerebellum	Molecular layer		●●●●●	NSP
	Purkinje cells	−	−	
	Internal granular layer nuclei	−		
	Internal granular layer neuropil		−	
	White matter and glial cells	−	−	
Cerebrum	Neuronal bodies	−	●●●●	Limited to neuronal bodies
	Neuropil	−	−	
	White matter	−	−	
CNS overall	Ependymal cells	−	●●●●●	Apical dot-like pattern
	Choroid plexus	●●●●●	●●●●●	NSP
Adrenal	Cortex	●●●●●	●●●●●	Lipid droplets are not stained
	Medulla	●	−	NSP
**Epithelial Cells**				
Esophagus	Squamous epithelial cells	●●●	●●●●●	NSP
Stomach	Foveolar cells	−	●●●●●	Apical perinuclear dot-like pattern
	Chief cells	−	●●●●●	NSP
	Parietal cells	−	−	NSP
Duodenum	Villous lining cells	−	●●●●	Apical perinuclear dot-like pattern
	Brunner’ ’s gland	−	●●	Perinuclear dot-like pattern
Ampulla of Vata	Glandular lining epithelium	−	●●●●●	Apical perinuclear dot-like pattern
Appendix	Lining epithelium	−	●●●●●	Apical perinuclear dot-like pattern
Colon	Lining epithelium	−	●●●●●	Apical perinuclear dot-like pattern
Pancreas	Ductal lining epithelium	−	●●●●●	Apical perinuclear dot-like pattern
	Exocrine cells	●●●	●●●●●	NSP
Liver	Intrahepatic duct lining epithelium	−	●●●●●	Strong staining at luminal border
	Hepatocytes	−	●●●●●	NSP
Salivary gland	Intercalated duct and striated duct	●	●	Perinuclear dot-like pattern
(parotid and	Serous secretory cells	●●	●●	NSP
submandibular)	Mucin secretory cells	−	●●●●●	Apical perinuclear dot like pattern
Lung	Bronchial lining cells	−	●●●●●	Apical perinuclear dot-like pattern
	Alveolar pneumocytes	●●	●	NSP
Breast	Lobular luminal cells	−	●●●●●	Apical cytoplasmic staining pattern
Kidney	Mesangial cells	−	●●●●	Perinuclear dot-like pattern
	Proximal tubules	−	●●●●●	NSP
	Distal tubules	−	●●●	Apical perinuclear dot-like pattern
Thyroid gland	Follicular epithelial cells	−	●●●●	Apical perinuclear dot-like pattern
**Other Cells**				
Tonsil	Lymphoid cells	●●●	●●●●	NSP
Spleen	Sinusoid lining cells	●●	●●●	Perinuclear dot-like pattern
Lung	Alveolar macrophages	●●	●●●●●	NSP

***N/A***, Not Applicable; ***NSP***, No specific staining pattern; −, Negative staining; 5 balls, 100% immunoreactivity; 4 balls, 75–100% immunoreactivity; 3 balls, 25–75% immunoreactivity; 2 balls, 5–25% immunoreactivity; 1 ball, <5% immunoreactivity.

#### Epithelial cells

**Figure 5:**
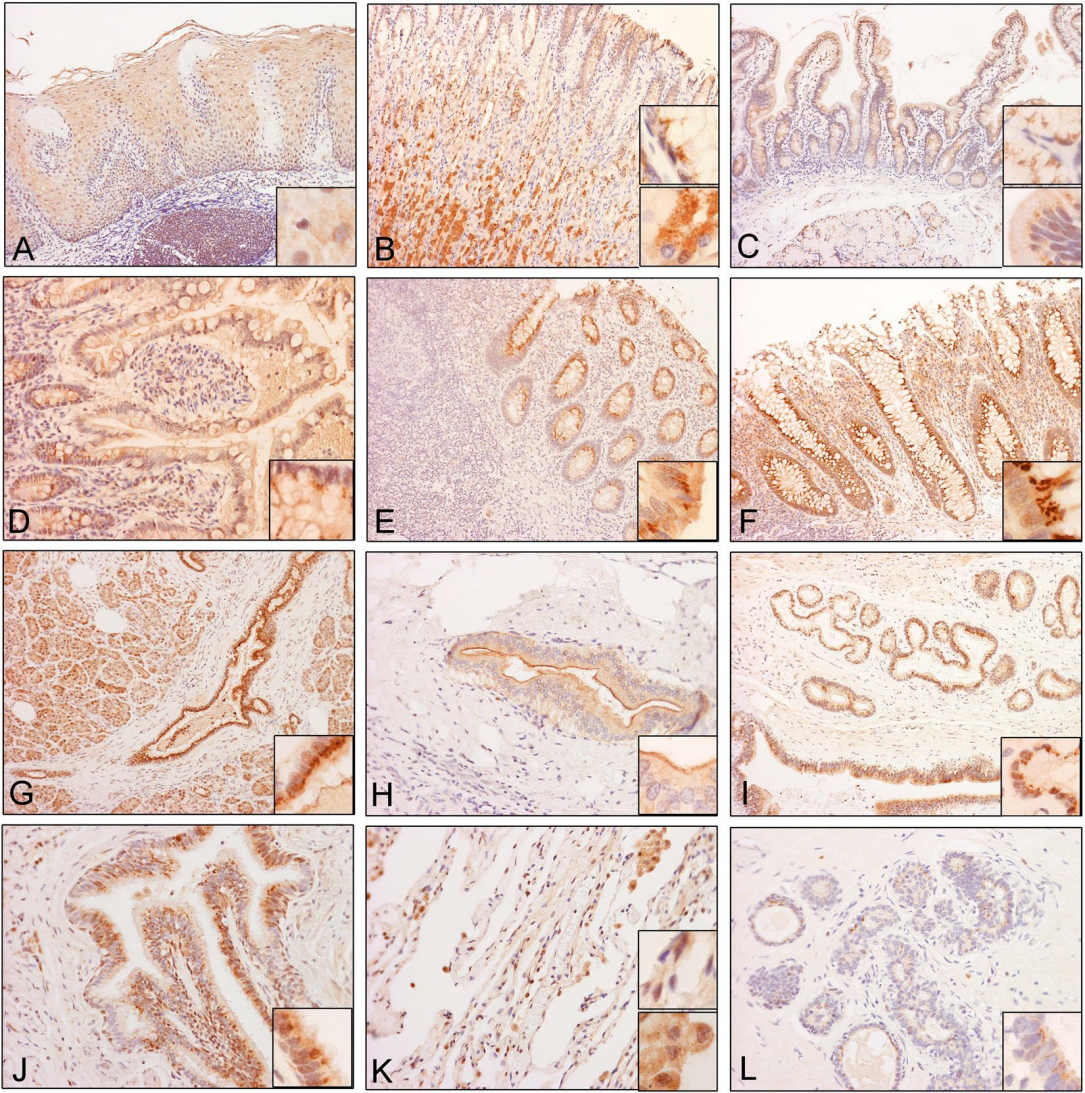
RD3 cellular localization in human tissues. Representative microphotographs showing RD3 expression and cellular localization in human (**A**) esophagus, (**B**) stomach, (**C**) duodenum, (**D**) small intestine, (**E**) appendix, (**F**) colon, (**G**) pancreatic duct, (**H**) intrahepatic duct, (**I**) ampulla, (**J**) bronchus, (**K**) lung, and (**L**) breast tissues.

**Figure 6:**
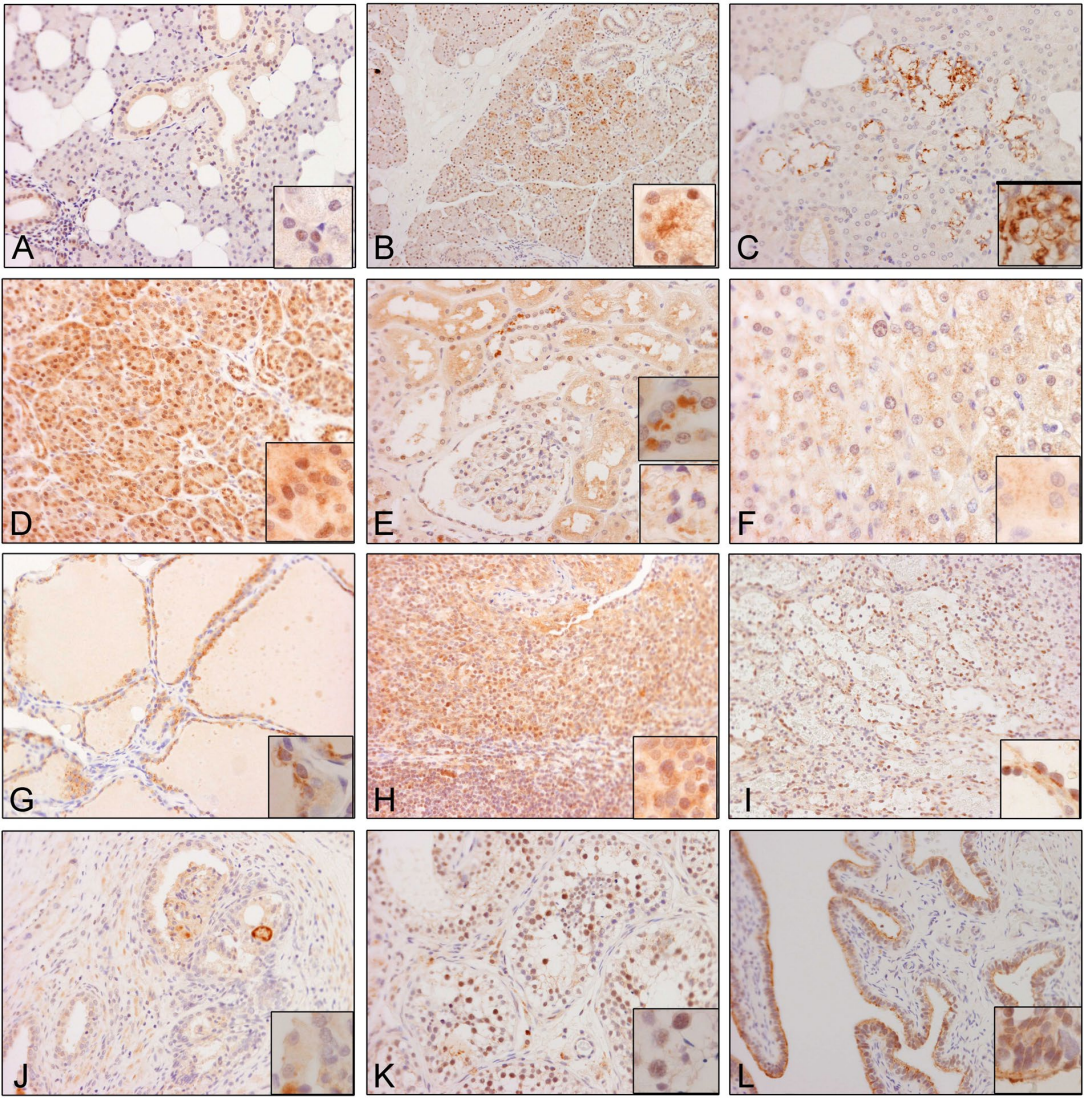
RD3 cellular localization in human tissues. Representative microphotographs showing RD3 expression and cellular localization in human (**A**) parotid gland, (**B**) submandibular gland, (**C**) salivary gland, (**D**) exocrine pancreas, (**E**) kidney, (**F**) liver, (**G**) spleen, (**H**) tonsils, (**I**) thyroid, (**J**) prostate, (**K**) testis, and (**L**) fallopian tube tissues.

The present study revealed strong expression of RD3 in epithelial cells of different organs. Results are summarized in Table 1 and illustrated in Figs 5 and 6.

#### Lining epithelial cells

In the squamous epithelium of esophagus, there were weak and heterogeneous immunoreactivities in the nuclei and moderate, widespread immunoreactivity in the cytoplasm (Fig. 5A). In the columnar epithelial lining of the gastrointestinal tract, hepatobiliary tree, pancreatic ducts, and bronchial tree, there was rather homogeneous and widespread cytoplasmic expression, but no nuclear immunoreactivity (Fig. 5B to J). Among the areas with moderate to high expression, the immunoreactivities were often in the form of dots, such as the positive structures in the apical areas (insets in Fig. 5B,C,D,E,F,G,I and J). The positive immunoreactivity at the luminal border of intrahepatic duct is a less common pattern (inset in Fig. 5H). The ducts of the salivary gland, where expression was weak in both nuclei and cytoplasm (Fig. 6A–C), and the alveolar epithelial cells lining the pulmonary alveoli (Fig. 5K), which demonstrate only nuclear immunoreactivities, were exceptions to the general rule. Macrophages in the lung were strongly positive for both nuclear and cytoplasmic immunoreactivity.

#### Secretory epithelial cells

In contrast to the homogeneous expression of cytoplasmic immunoreactivities in the lining epithelial cells, the secretory epithelial cells demonstrated variable immunoreactivities. In stomach tissues, the surface foveolar cells showed abundant, yet moderate, RD3 positivity selectively in the cytoplasm (see top insert), while the parietal cells and mucus neck cells were almost negative for RD3. However, the zymogenic chief cells displayed a strong and abundant cytoplasmic positivity (Fig. 5B). Serous secretory cells in both parotid and submandibular glands were only weak to moderately immunoreactive (Fig. 6A,B). However, mucin-secreting cells were strongly immunoreactive (Fig. 6C and inset). In addition, exocrine pancreatic cells were strongly immunoreactive (Fig. 6D).

Strong apical dot-like immunoreactivities similar to that observed in the intestinal epithelial cells were also noted in the distal tubules, but not in the proximal tubules. Perinuclear dot-like immunoreactivities were observed in the mesangial cells in renal glomeruli (Fig. 6E). Weak, but extensive, immunoreactivities were seen in hepatocytes (Fig. 6F). Dot-like immunoreactivity was also found in the thyroid epithelium.

#### Other Cell Types

There was widespread immunoreactivity among lymphoid cells in the tonsil (Fig. 6H). However, we observed that immunoreactivity in lymphoid tissue is variable in different organs. The sinusoidal lining cells in the spleen (Fig. 6I) were positive for RD3. Alveolar macrophages in the lung were also strongly positive (Fig. 5K).

## Discussion

In the present study, we characterized RD3 expression across normal human tissues using an antibody suitable for FFPE tissue analysis. The antibody explicitly detects RD3 protein, as evident from its immunoreactivity both in a panel of neuroblastoma cell lines and in a panel of human normal tissues (Fig. 1). Moreover, our results from peptide and scrambled peptide blocking studies on the panel of human tissues clearly portrayed the specificity of the Ab with no non-specific cross reactivity. Although it is insignificant, on a caution note, the disappearance of very faint bands other than RD3 in the presence of neutralizing peptide could indicate the possibility of some residual cross-reactivity in tissues. However, comparative analysis of RD3 localization in retinal layers with a panel of six commercially available antibodies raised against various regions of human RD3 protein, clearly demonstrated the specificity and efficiency of the custom synthesized Ab (Figure S5). It is relevant to mention that the subcellular distribution patterns of RD3 in retinal tissue observed in this study did not completely agree with the previously documented localization patterns^1,10^. To that note, previously reported RD3 antibodies are either raised against mouse (except human RD3 #497 etc.) or the immuno-localization patterns are assessed only in mouse (wild type and or RD3 mice) retina^1,10^. Also, studies have indicated the advancement of antibody synthesis resulted in the betterment of RD3 labeling from only retinal photoreceptor outer segment (POS) localization to the better revealing of localization in POS, inner segment, axoneme and outer plexiform layer^10^, and agrees at least in part with the current observation. To our knowledge this is the first report of RD3 expression and localization in normal human tissues. Markedly, our results showing a single 23-kDa band in normal human retina demonstrated the specificity of the synthesized RD3 antibody and validate the relative abundances of RD3 expression in other human normal tissues. Consistently, our results comparing the RD3 transcript levels (QPCR analysis) in human retina, colon, pancreas, submandibular, lungs and duodenum identified not only the abundance of RD3 transcripts in human normal tissues but also indicated the relative expression patterns of RD3 in these tissues compared to human retina. The mRNA expression patterns well corroborated to the RD3 protein expression patterns assessed with the immunoblotting as well with IHC.

In silico data analysis of RD3 transcription in wide array of clinical tissues recognized the basal as well as inter- and intra-tissue-specific fluctuations in RD3 transcription in normal human tissues. Our survey of the expression of RD3 in normal human tissue led to three major novel findings. First, RD3 is strongly expressed, typically in periapical dot-like immunoreactivities, in lining epithelial cells that range from choroid plexus cells to epithelial cells lining the gastrointestinal tract and hepatobiliary tract. This phenomenon extends to ependymal cells, which are the lining cells of the ventricles of the central nervous system and have a partial epithelial phenotype. Second, although RD3 is strongly expressed in retinal cells, which are basically neurons, it is only weakly immunoreactive or negative in neuronal bodies and their process in the central nervous system. Third, although RD3 is strongly immunoreactive in some subsets of neuroblastomas and neuroblastoma cell lines, it is negative in the adrenal medulla, from which most adrenal neuroblastomas originate. These findings are consistent with the earlier studies describing the presence of RD3 mRNA in different tissues (see Fig. 2 *in silico* data analysis). Based on these observations, we believe that RD3 has an important role in the normal functioning of epithelial cells. Interestingly, no evidence of any malfunction other than eye degeneration was documented, at least thus far, in RD3 mice. However, negative RD3 staining in the adrenal medulla observed in this study raises questions regarding its role in normal adrenal embryonal genesis and maturation, in addition to its role in adrenal neuroblastomas.

Earlier, we characterized the expression of RD3 in human neuroblastoma cells; the expression of RD3 is highly restricted in metastatic site-derived aggressive cells. Determining the functional role of any molecular candidate in cancer biology, genesis, and progression requires a better understanding of its presence and abundance under healthy conditions. Information on mere mRNA status (through high throughput platforms) may not directly reflect the levels of constitutive functional protein status due to mRNA degradation by miRNAs/siRNAs^11^, mRNA splicing errors (exon skipping, failure to remove intron), and defects/regulations in translational machinery (amino-acid misincorporation, tRNA misacylation, premature termination, read-through, frameshift)^12–14^. Hence, it is critical to recognize protein expression and cellular localization along with gene transcriptional status. Furthermore, considering the tissue-specific responses of cancer genesis and progression, it is crucial to understand tissue- and/or cell- specific baseline transcription and protein expression/localization. For the first time, the results presented here demonstrate the constitutive expression of RD3 in numerous normal human tissues.

Studies have revealed the influential genetic defects of the RD3 gene in photoreceptor degeneration, heavily contributing to early-onset (childhood) blindness^15^. To that end, the RD3 gene is highly expressed in the retina^3^, and reveals increasing expression through early postnatal development. Although mutation-associated loss of RD3 protein^3^ has been causally linked to early stage retinal degeneration^16^, a baseline abundance of RD3 in normal tissues has not been documented. However, we recently showed that RD3 is significantly lost both at the mRNA and protein levels in a high-risk progressive childhood tumor, neuroblastoma^8^. Further, we demonstrated that RD3 regulates the metastatic state and potential of tumor cells^8^. These findings signify the influential molecular functions of RD3 protein and demonstrated that RD3 protein loss either by genetic defects or ongoing acquisition of molecular events could lead to pathogenesis. To that end, RD3 protein includes a putative coil-coil domain (that serves as a protein-interaction site) and a number of conserved sites for protein modification, including phosphorylation and sumoylation. In any event, the association of RD3 loss with the high-risk disease in multiple cohorts of neuroblastoma patients and RD3 protein influencing the regulation of tumor cell migration, invasion, and tumorosphere formation validates the causal role of RD3 protein in tumor progression^8^.

We obtained reliable staining of FFPE tissues using a custom-synthesized RD3 antibody. Similar cell types from the same and different tissues demonstrated varied staining intensities and patterns. Staining was observed in the cytoplasm and/or the nucleus, consistent with earlier investigations^3,17,18^, ruling out any non-specific or fixation artifacts. Furthermore, researchers have shown that nuclear localization of RD3 is associated with promyelocytic leukemia-gene-product (PML) bodies^3^. The different patterns of cellular staining support several roles for RD3. Nuclear co-localization of RD3 with PML might indicate a role in the regulation of tumor progression, while its cytoplasmic location may be involved in other critical cellular functions.

The organ distribution of RD3 protein has never been characterized. In the present study, we reported the organ distribution and cellular localization of RD3 in human lungs, bile duct, pancreas, small intestine, stomach, submandible, appendix, cerebellum, colon, small bowel, kidney, liver, parotid, placenta, thyroid, tonsil, breast, duodenum, esophagus, fallopian tube, hypothalamus, lymph node, olfactory bulb, prostate, salivary gland, skin, spinal cord, spleen, testes, thalamus, thymus, and uterus tissues. We observed significant strong cell-specific nuclear and cytoplasmic localization in human retina, consistent with earlier studies^3^. Comparable or higher RD3 expression was observed in lungs and GI tissues, while other tissues exhibited moderate RD3 positivity (see Fig. 3). However, staining varied between systems, organs, and cell types, indicating that RD3 has a tissue-specific and/or function-specific role.

Although the mechanism by which RD3 loss mediates retinal degeneration has been extensively documented^1,2,4–6^, the mechanism(s) involved in RD3 loss and loss-associated tumor progression are unknown. Our findings demonstrate that, in normal tissues, RD3 has various sub-cellular locations and a heterogeneous pattern of expression. These results provide a critical platform that will allow us to delineate RD3-associated and/or driven mechanisms. One could argue that understanding the basal levels of RD3 in human fetal tissues would be relevant to defining the role of RD3 in early childhood diseases, including neuroblastoma. In addition, identifying the RD3 protein signature in cancer tissues is required to understand its role in cancer biology. We acknowledge these limitations; however, to appreciate the outcomes of any such studies, the understanding of RD3 expression in normal adult human tissues, which is characterized in this study, is critical.

## Materials and Methods

### In silico transcriptomics analysis

Three independent database portals, (i) Genotype-Tissue Expression (GTEx), (ii) Gene Expression across Normal and Tumor tissue (GENT), and (iii) Medisapiens *in silico* transcriptomics online (IST), were used to study the mRNA expression of RD3 across normal human tissues and cells. The samples, 53 healthy tissues (total *n* = *8232*) included in the GTEx database, were analyzed on the Affymetrix and Illumina platforms and expressed in calculated RPKM with isoforms collapsed to a single gene with no other normalization steps. The GENT database contained expression analysis in 25 healthy human tissues (total *n* = *3210*) from the Affymetrix platform. The IST database included data analysis of gene expression from the Affymetrix platform across 49 healthy tissues (total *n* = *1706*) with unique normalization and data quality verifications, allowing the gene expression profiles collected from different studies to be combined to generate an overview of the expression profile in human tissues.

### Tissue specimens and immunohistochemistry (IHC)

Normal human tissue blocks were retrieved from the formalin-fixed, paraffin-embedded (FFPE) archival materials at the Department of Pathology, University of Oklahoma Health Sciences Center. RD3 expression and localization was investigated in different tissues, including retina, central nervous system (brain, spinal cord, olfactory bulb), gastrointestinal tract (esophagus, stomach, duodenum, appendix, colon), pancreatic hepatobiliary tract (parotid and submandibular gland, liver, intra-hepatic duct, pancreas), and other organs (lung, kidney, placenta, uterus, thymus, prostate skin, fallopian tube, thyroid, tonsil, breast). All protocols were approved by the University of Oklahoma Health Sciences Center Institutional Review Board with permission for the research use of de-identified control specimens collected for diagnostic purposes. All experiments were performed with University of Oklahoma Health Sciences Institutional Review Board for the protection of human subjects guidelines and regulations. All tissue section processing and immunohistochemistry (IHC) was performed in the COBRE-Cancer Tissue Pathology Core located at the University of Oklahoma Stephenson Cancer Center, as described in our earlier studies^8,19,20^. IHC for RD3 (customized antibody, 4.0 μg/ml) was performed utilizing an automated IHC machine (Leica Bond III) according to the manufacturer’s protocol using the Bond™ Polymer Refine detection system. A peroxidase-diaminobenxidine visualization process was employed, which gave positive immunoreactivity a brown color. Appropriate tissue histology controls stained with hematoxylin-eosin stain and negative controls with isotype matched (rabbit IgG Isotype control, ThermoFisher Scientific, Rockford, IL) no primary antibody (Figure S4) were examined in parallel. The slides were digitally scanned into virtual slides using an Aperio Scan Scope (Aperio Technologies, Inc., Buffalo Grove, IL, USA) slide scanner at 20x magnification. The whole slide images were then group-analyzed for RD3-specific positivity using Aperio image analysis and quantification software (Aperial Tool Box) with the appropriate algorithms for IHC. Parameters for analysis included cytoplasmic, nuclear, and total staining intensity. Automated strong positivity was quantified in multi-sections and/or multi-slides for each tissue type using RD3-specific (cytoplasmic and nuclear) image analysis algorithms. The tissue-specific metadata were exported to Excel. Tissue-specific expression was profiled by comparing with no primary antibody (Ab) controls, and the means and *SD* were plotted (GraphPad Prism). Manual interpretations regarding cell type-specific localization and subcellular localization were performed by two anatomic pathologists (KMF and KLK). Still images were taken with a conventional light microscope (Nikon Eclipse 80i) equipped with a digital camera. The intensity of immunoreactivity in different cellular locations was scored with a 3-tier system, and the extent of immunoreactivity was scored with a 5-tier system, as detailed in the legend of Table 1. To compare the RD3 IHC labeling specificity and efficiency of the custom synthesized Ab, IHC labeling was compared with commercially available RD3 Abs raised against various regions of human RD3 protein. For this, sections of human retina were immunostained with rabbit polyclonal RD3 Abs against epitope mapping between AA 52-112, AA 7-67 (both Abs from Antibody Verify), AA 36-85 (from Abcam) and; mouse monoclonal Abs raised against epitope mapping between AA 62-87, AA 145-175 and, AA 135-194 (all Abs from Santa Cruz Biotechnology Inc.). A negative control with no primary Ab is also included.

### Cell Culture

The human neuroblastoma (SH-SY5Y, SK-N-AS, SK-PNDW, and IMR-32) cells were obtained from ATCC (Manassas, VA) and were cultured and maintained as described in our earlier publications^21^. In brief, SH-SY5Y cells were maintained as monolayer cultures in DMEM/F-12 50/50 (Mediatech, Inc., Herndon, VA) supplemented with 1.5 g/L sodium bicarbonate, 2 mm l-glutamine, 1% nonessential amino acids, 1% minimum essential medium vitamins, 5000 IU/ml penicillin, 5000 μg/ml streptomycin, 1% sodium pyruvate, and 10% FBS (Invitrogen). Human SK-N-AS, IMR-32, and SK-PN-DW cells were maintained in Dulbecco’s modified eagle’s medium supplemented with 0.1 mM nonessential amino acids, 1.5 g/L sodium bicarbonate, 5000 IU/ml penicillin, 5000 g/ml streptomycin, 0.011% sodium pyruvate, and 10% FBS. For passage and for all of the experiments, the cells were detached using trypsin (0.25%) and EDTA (1%), re-suspended in complete medium, counted (Countess; Invitrogen), and incubated in a 95% air, 5% CO_2_ humidified incubator.

### Plasmid preparation and DNA transfection

RD3 plasmid preparation and DNA transfection was performed as described in our earlier studies^8^. Expression of RD3 (Human retinal degeneration 3, transcript variant 1, Origene) was conducted with TurboFectin 8.0 reagent (Origene).

### Immunoblotting

Total protein extraction and immunoblotting were performed as described in our earlier studies^21,22^. For this study, immunoblotting was performed in the protein lysates (50 μg) of human neuroblastoma (SH-SY5Y, SK-NA-AS, SK-PN-DW, IMR-32) cells; normal human retina, duodenum, pancreas, lungs, colon, submandibular glands, brain and spinal cord and; in lysates from RD3 expressing SH-SY5Y cells, RD3 null SH-MSDAC cells and, ectopically RD3 re-expressed SH-MSDAC cells. The protein-transferred membranes were incubated with human RD3 antibody and were developed with the appropriate anti-rabbit secondary Ab (Bio Rad Laboratories, Hercules, CA). For peptide competition assay, the RD3 antibody was neutralized by mixing antibody with antigen (peptide) prior to immunoblotting. For defining the specificity of the peptide antigen competition, lysates from identical set of human tissues were immunostained with neutralized Ab with the scrambled sequence (SPDLRRESWDPVETP, synthesized by GenScript, Piscataway, NJ on our initiative) peptide containing same amino acid content. Blots were stripped and reblotted with mouse monoclonal α-tubulin (Santa Cruz Biotechnology, Inc., Dallas, TX) or mouse monoclonal GAPDH (EMD Millipore, Billerica, MA) confirm equal loading of the samples. Specificity and efficiency of RD3 labeling was scrutinized using full length blots. For this study, broad-range blue pre-stained protein standard (New England Biolabs, Ipswich, MA, USA), was used for observing protein separation, transfer efficiency and for verification of protein size. Since the molecular standards do not show up in the chemiluminescent exposure, the ladder is not included in the pictures. Band intensity quantification was performed using Quantity One (Version 4.6.5, Bio Rad) 1D image analysis software and were plotted/analyzed with GaphPad PRISM (Version 7.03, GraphPad Software, Inc., La Jolla, CA).

### Quantitative Real-time PCR

Total RNA extraction and RD3 gene transcript levels were investigated as described earlier^8,23^. We used b-actin as a positive control. A negative control without template RNA was also included. Each experiment was carried out in triplicate. The DDCt values were calculated by normalizing the gene expression levels to b-actin. The relative expression level was expressed as fold change.

## Electronic supplementary material

Supplementary Figures S1–S6
