# Supplementary material for: Retinal Degeneration Protein 3 (RD3) in normal human tissues: Novel insights
Source: Sci Rep. 2017 Oct 13;7:13154. doi: 10.1038/s41598-017-13337-9 (PMC5640666; doi:10.1038/s41598-017-13337-9)
Supplement: Supplementary file 1 — Supplementary Figures S1–S6 [file 41598_2017_13337_MOESM1_ESM.doc]

Retinal Degeneration Protein 3 (RD3) in normal human tissues: Novel insights.

Sheeja Aravindan^1#^, Dinesh Babu Somasundaram^2#^, Kwok Ling Kam^3#^, Karthikeyan Subramanian^2^, Zhongxin Yu^3^, Terence S. Herman^1,2^ Kar-Ming Fung^1,3^, and Natarajan Aravindan^2,3*^

**
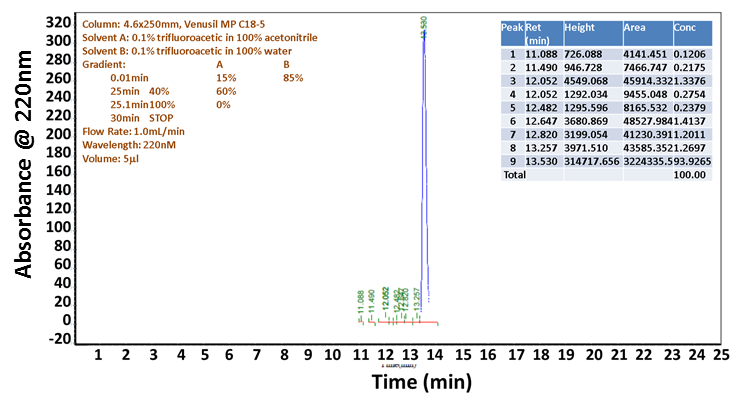

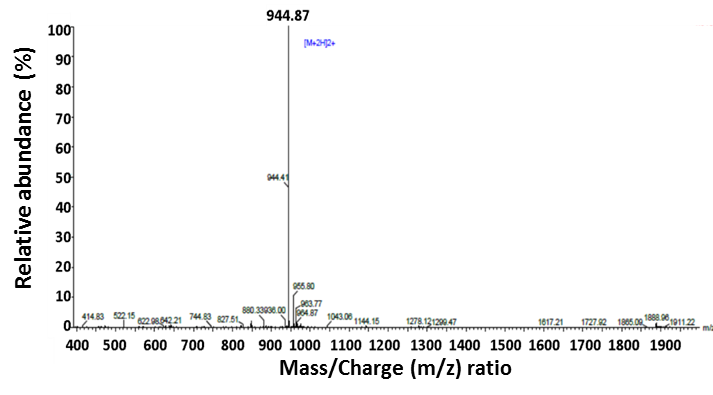

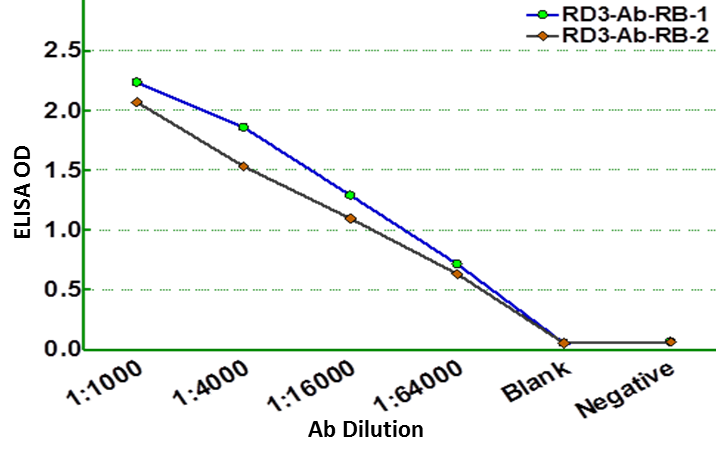
**

**Absorbance @ 220nm**

**(A)**

**(B)**

**(C)**

**Figure S1. *Antibody validation:*** Characterization of customized sequence specific (amino acids 171-183) RD3 antibody produced by the NeoBioLab (Cambridge, MA) on our initiative. **(A)** Chromatogram showing the of HPLC profile of custom synthesized RD3 antibody. Mobile phase consisted of a gradient system of acetonitrile and water (0.1% trifluoroacetic acid) at a flow rate of 1 mL/min. The stationary phase consisted of a Venusil MP C18 -5 column (250 x 4.6 mm), and the UV signal was recorded at 220 nm. RD3 Ab spiked (~94%) at a retention time of 13.53 minutes. **(B)** Mass spectrometry analysis of customized RD3 antibody showing relative abundance (100%) of RD3 Ab with a m/z ratio of 944.87. **(C)** ELISA analysis showing robust and concentration dependent specificity of custom synthesized RD3 Ab, with a definite and measurable sensitivity in as low as 1:64000 dilutions.

**Figure S2.** ***RD3 Ab specific labeling – peptide competition:*** Representative microphotographs showing RD3 antibody-specific labeling in human colon FFPE tissue sections. No primary Ab controls were included. Automated IHC with RD3 Ab demonstrated the sub-cellular selectivity (predominantly localized in the nucleus and perinuclear area, with weak to moderate cytoplasmic positivity) and specific RD3 immunoreactivity (strong, moderate, and weak positivity) in colon tissues. Ab premixed with peptide (Ab neutralization) showed complete blocking of RD3 staining, revealing antibody-specific IHC staining in FFPE sections.


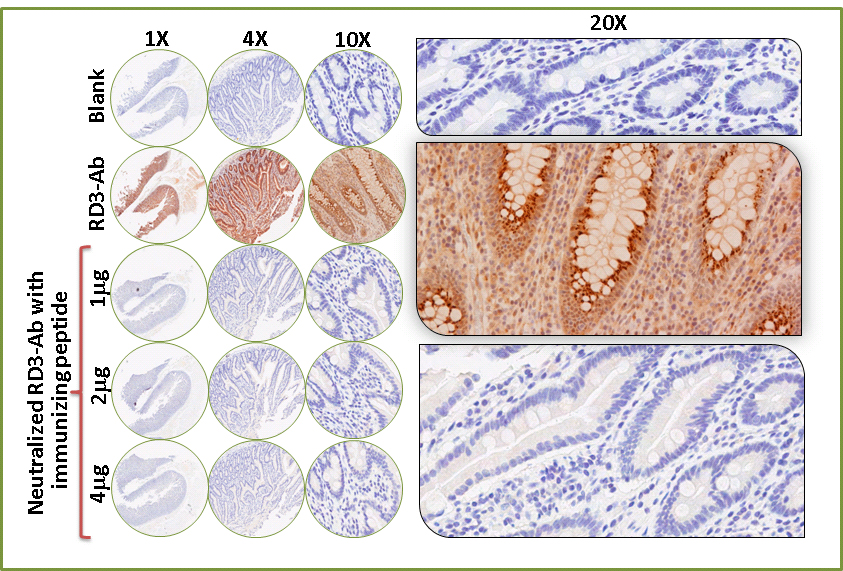


**
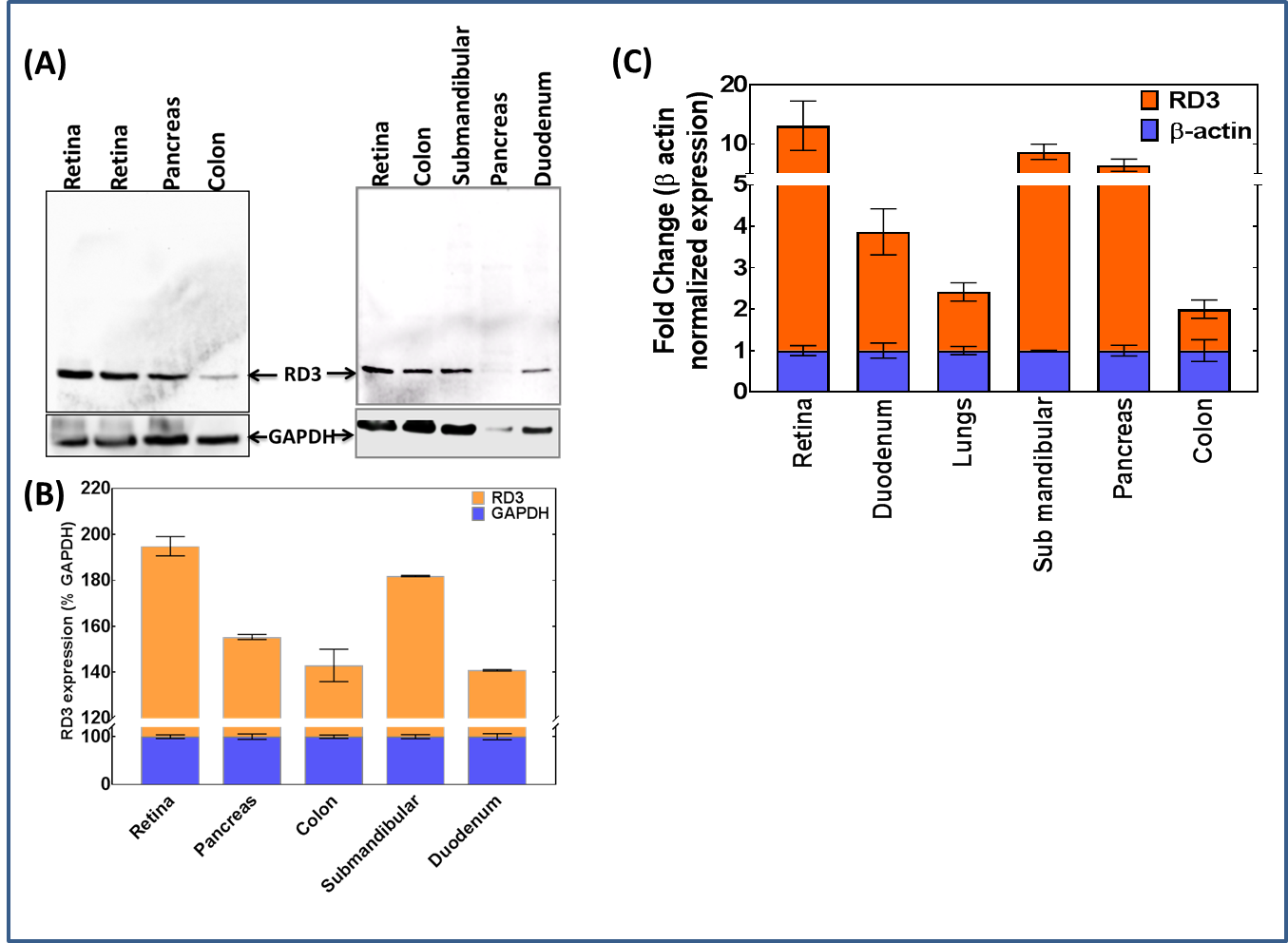
**

**Figure S3. (A-B) RD3 antibody validation and abundance in normal tissues: (A)** Immunoblot showing RD3 specific labeling by the custom synthesized antibody without any non-specific events. Cell lysates from human retina, pancreas, colon, submandibular and duodenal tissues blotted with synthesized RD3 antibody resulted in the labeling of single strong band at 23 kDa. Full-length blots are presented in Supplementary Figure S6. **(B)** Histograms of Quantity one band ID gel analysis showing relative expression levels of RD3 in human retina, pancreas, colon, submandibular and duodenum. **(C) RD3 transcription in normal human tissues:** Histograms from QPCR analysis showing transcriptional modulations of RD3 in human retina, pancreas, colon, submandibular and duodenum. Expression of RD3 was normalized to β-actin expression in corresponding tissues and is presented as fold change.

**Figure S4. *Isotype matched controls:*** Representative microphotographs showing no non-specific labeling with isotype matched controls in human **(A) r**etina, **(B)** cerebrum, **(C)** colon, **(D)** liver, **(E)** lungs, **(F)** breast, **(G)** testis, **(H)** tonsils and, **(I)** pancreas. IHC was performed under similar conditions as discussed, however replacing RD3 Ab with rabbit IgG isotype control. [Magnification 20x; Insert, 60x]


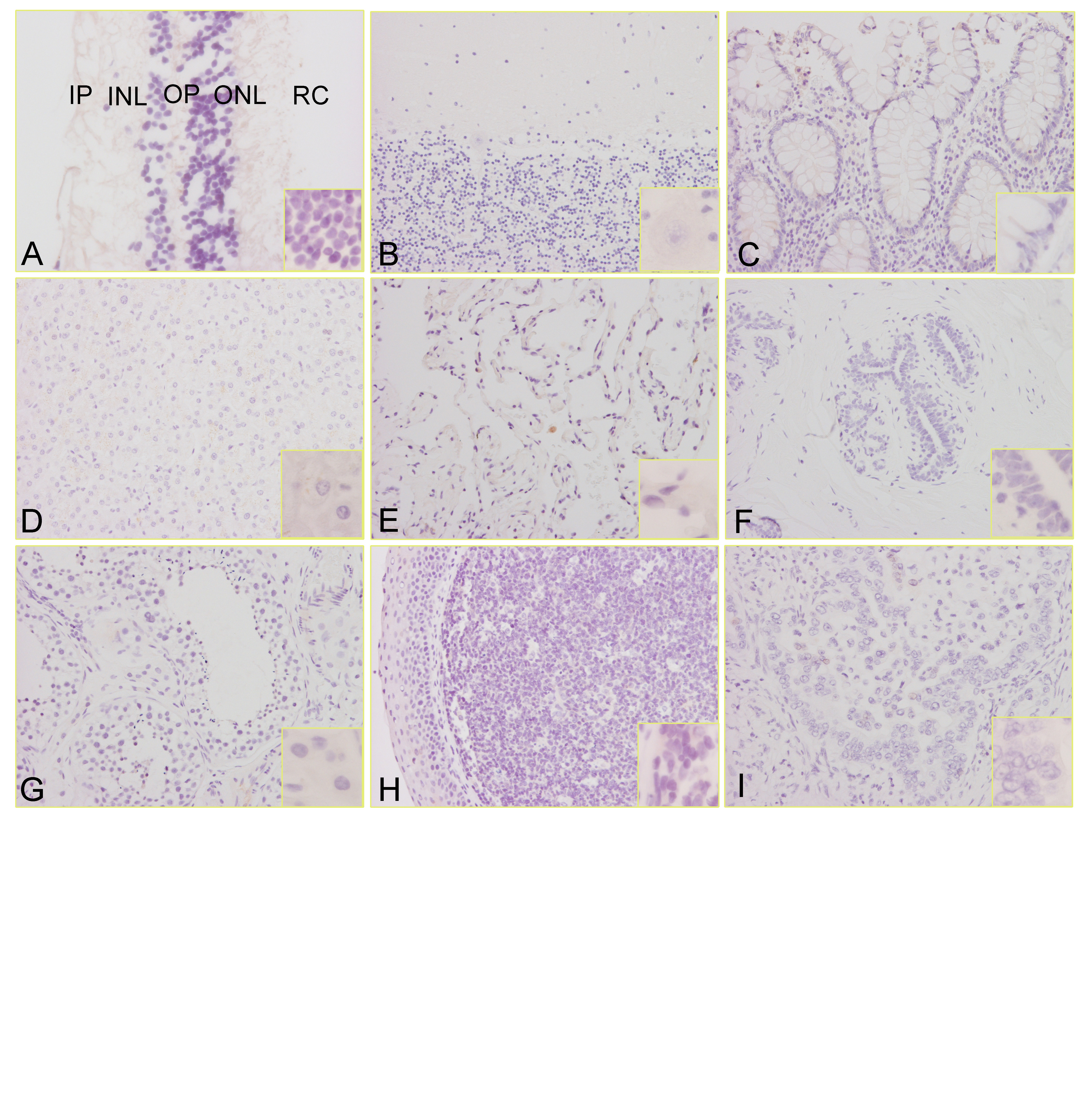


**Figure S5. *Custom synthesized RD3 Ab efficiency and specificity comparison*:** Representative microphotographs showing RD3 staining patterns in human retina with **(A)** custom synthesized antibody utilized in this study in comparison with commercially available rabbit polyclonal Abs against epitope mapping between **(B)** AA 52-112, **(C)** AA 7-67 (both Abs from Antibody Verify) ; mouse monoclonal Abs raised against epitope mapping between **(D)** AA 62-87, **(E)** AA 145-175, **(F)** AA 135-194 (all Abs obtained from Santa Cruz Biotechnology Inc.) and**;** **(G)** rabbit polyclonal RD3 Ab epitope mapping between AA 36-85 (from Abcam). A negative control with no primary Ab is also included **(H).** Relatively, custom synthesized RD3 Ab utilized in this study showed immaculate labeling specificity and efficiency in human retina.


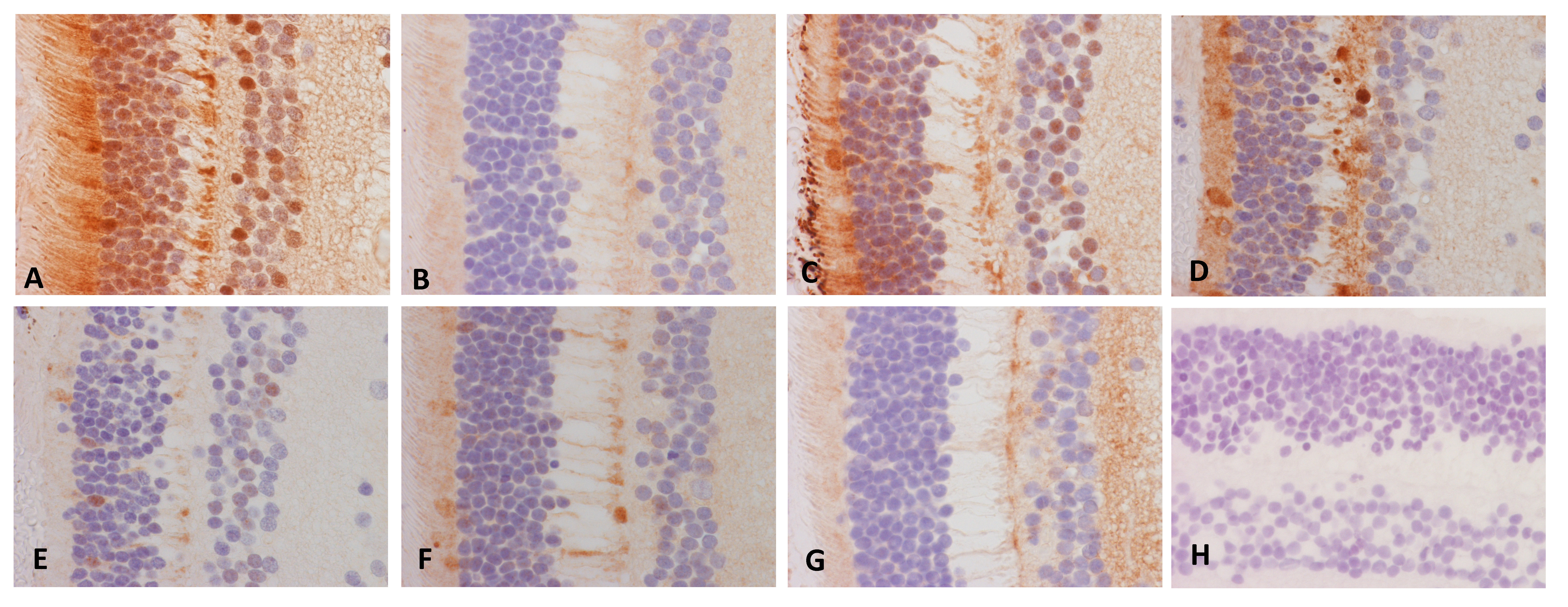


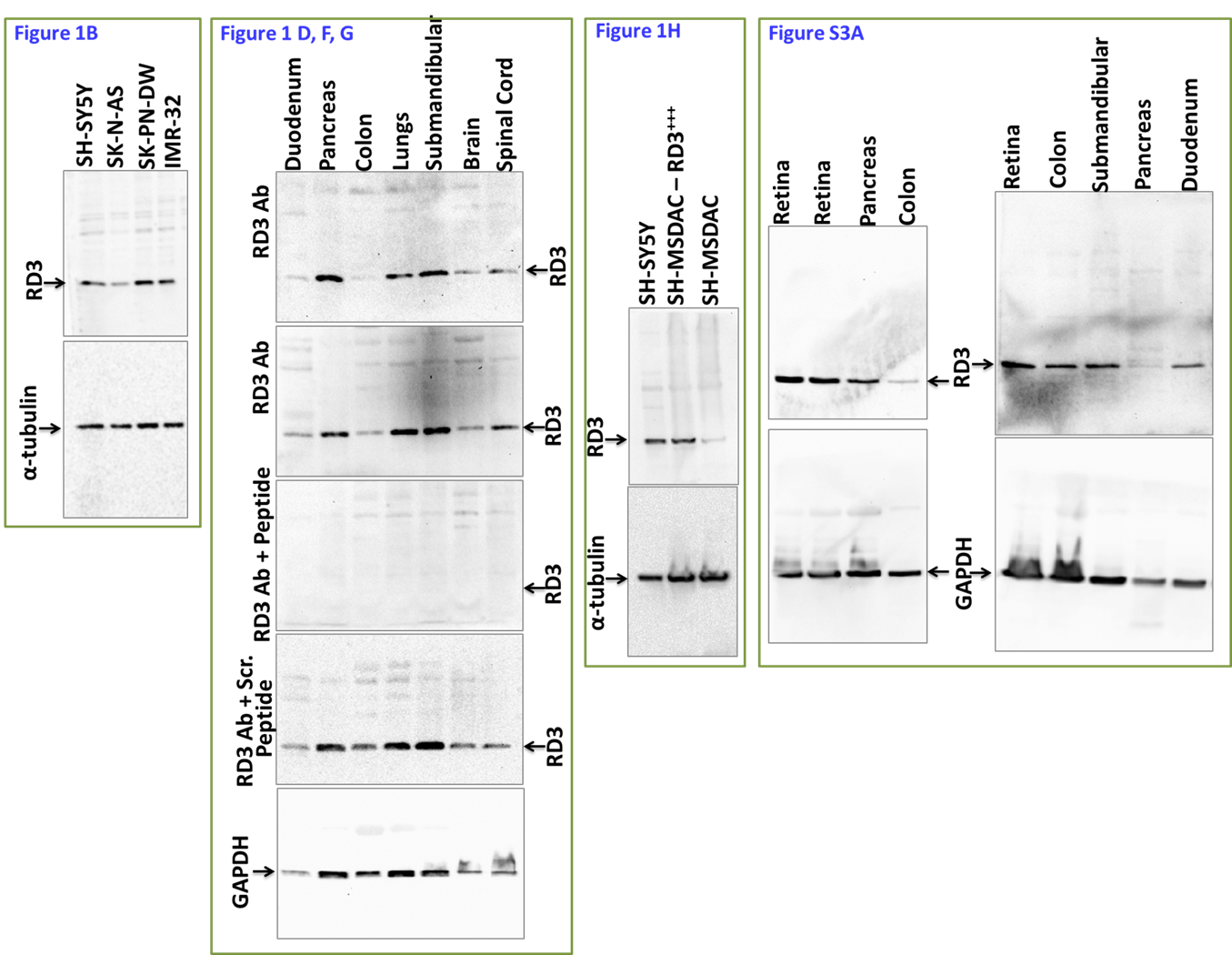


**Figure S6.** Full length blots for the figures presented in Figure 1 and Supplementary Figure S3.
